# Effects of Melatonin Treatment of Postharvest Pear Fruit on Aromatic Volatile Biosynthesis

**DOI:** 10.3390/molecules24234233

**Published:** 2019-11-21

**Authors:** Jianlong Liu, Hanting Liu, Ting Wu, Rui Zhai, Chengquan Yang, Zhigang Wang, Fengwang Ma, Lingfei Xu

**Affiliations:** 1 College of Horticulture, Qingdao Agricultural University, Changcheng Road No.700, Qingdao 266109, Shandong Province, China; liujianlong1357@163.com; 2 College of Horticulture, Northwest A&F University, Taicheng Road NO.3, Yangling 712100, Shaanxi Province, China; hylht3598@163.com (H.L.); 18829351538@163.com (T.W.); zhongdishaonian@sina.com (R.Z.); cqyang@nwsuaf.edu.cn (C.Y.); wzhg001@163.com (Z.W.); fwm64@nwsuaf.edu.cn (F.M.)

**Keywords:** pear, melatonin, aroma, enzyme activity, gene expression

## Abstract

Aroma affects the sensory quality of fruit and, consequently, consumer satisfaction. Melatonin (MT) is a plant growth regulator used to delay senescence in postharvest fruit during storage; however, its effect on aroma of pear fruit remains unclear. In this study, we assessed the effects of 0.1 mmol L^−1^ MT on volatiles and associated gene expression in the fruit of pear cultivars ‘Korla’ (*Pyrus brestschneideri* Rehd) and ‘Abbé Fetel’ (*Pyrus communis* L.). MT mainly affected the production of C_6_ aromatic substances in the two varieties. In ‘Korla’, MT inhibited expression of *PbHPL*, and reduced hydroperoxide lyase (HPL) activity and content of hexanal and (*E*)-hex-2-enal. In contrast, MT inhibited activity of lipoxygenase (LOX), reduced expression of *PbLOX1* and *PbLOX2*, promoted *PbAAT* gene expression, increased alcohol acyltransferase (AAT) activity, and increased propyl acetate, and hexyl acetate content in ‘Abbé Fetel’ that similarly led to the reduction in content of hexanal and (*E*)-hex-2-enal. Content of esters in ‘Abbé Fetel’ pear increased with increasing postharvest storage period. Although mechanisms differed between the two varieties, effects on aroma volatiles mediated by MT were driven by expression of genes encoding LOX, HPL, and AAT enzymes.

## 1. Introduction

Melatonin (MT) (*N*-acetyl-5-methoxytryptamine) is a nontoxic biological molecule produced by the pineal gland in animals and in different tissues in plants. Studies have identified a range of functions of MT in plants, including its scavenging of reactive oxygen species (ROS) that improves resistance to biotic and abiotic stresses, such as pathogen attack, extreme temperatures, salinity, drought, waterlogging, and low-sulfur [1,2,3,4,5,6].

The shelf-life of postharvest produce declines with time, due to deterioration in quality associated with ripening and senescence processes. As a result, a range of treatments to maintain the quality and shelf life of postharvest fruit has been developed, including the application of MT that reduces ROS activity, increases production of antioxidant enzymes during postharvest storage, and inhibits ethylene synthesis to delay ripening and softening [7].Exogenous application of MT to grapes on the vine changes their polyphenol metabolism, carbohydrate biosynthesis, and, importantly, ethylene signaling [8] that, when reduced, has been shown to improve antioxidant activity in cassava fruit [9] and aroma, color, sugar content, and overall postharvest quality of tomato fruit [10].

Color, level of sugar-acid, and content of aromatic compounds are key quality indexes used in the climacteric pear fruit, and many studies have shown that aroma biosynthesis is dependent on the ethylene biosynthetic pathway. In climacteric fruit, ripening is characterized by an increase in respiration rate, and a greater production of volatiles and ethylene [11]. Ripening of apple fruit is largely driven by ethylene production that regulates the volatile ester biosynthetic pathways. Potential biochemical steps involved in the modulation of ester production under ethylene regulation have been demonstrated in a transgenic line of apple with high suppression of ethylene biosynthesis [12], and it has been shown that treatment of apple fruit with ethylene regulates volatile biosynthesis and gene expression [13]. Similarly, biosynthesis of ethylene in pear fruit is closely related to the production of aromatic volatile compounds. For example, application of the ethylene action inhibitor 1-methylcyclopropene (1-MCP) changed the aroma components in ‘Ruanerli’ and ‘Alexander Lucas’ pears [14].

Pear varieties comprise two types: those that are ready to eat immediately upon harvest, where ethylene production has no effect on quality, and those that require ripening and softening triggered by an increase in respiration and ethylene production. ‘Korla’ pear is an example of the former. It is one of the most popular fruits in China [15] and is exported to many countries around the world. ‘Abbé Fétel’ is an example of the latter, and is the most important pear variety cultivated in Italy. It is characterized by a strong aroma after softening, indicating it is suitable for consumption. Aromatic components of pear fruit are dominated by C_6_ aldehydes and alcohols that are generated by linoleic and linolenic acids as precursors through the lipoxygenase (LOX) pathway [16]. LOX and hydroperoxide lyase (HPL) convert linoleic and linolenic acids to hexanal and hexenal, respectively, via 9- and 13-hydroperoxide isomers, and aldehydes are then reduced to the corresponding C_6_ alcohols by alcohol dehydrogenase (ADH). Aroma esters are subsequently produced through alcohol acyltransferase (AAT) that catalyzes the final linkage of an acyl moiety and an alcohol. Although specific enzymes and genes involved in fatty acid-derived lactone (cyclic ester) formation have not been characterized [16], studies have shown that gene expression and enzyme activity are closely related to the synthesis of volatiles in fruit [11,17].

Here, we hypothesized that MT changes aroma components in pear fruit, because it inhibits the release of ethylene in postharvest fruit. We tested this hypothesis by analyzing the effects of MT on aroma components and indicators of the LOX pathway in two varieties of pear with contrasting ripening characteristics.

## 2. Results

### 2.1. Effects of MT on Volatile Production during Postharvest Ripening

**Figure 1:**
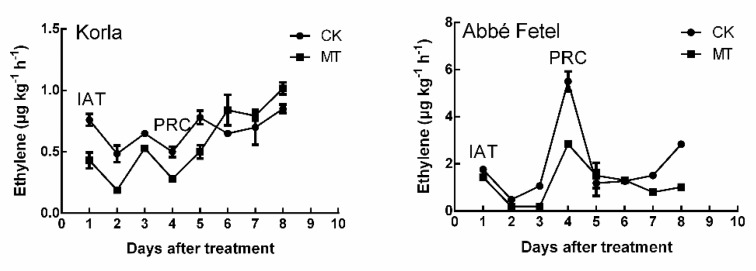
Effect of melatonin on ethylene production during storage. CK: negative control; MT: 100 μM melatonin; data are means of 5 replicates ± SD.

There was no difference in the aroma maps of ‘Korla’ IAT and at PRC (Figure S1a); however, substances were much richer at PRC than IAT for ‘Abbé Fetel’ (Figure S1b). MT inhibited ethylene production in the pears, and it was lower in ‘Korla’ than in ‘Abbé Fetel’ (Figure 1). In ‘Korla’, there was no significant ethylene release peak, but after the 4th day, the ethylene content in the fruits began to gradually increase, so we designated the fourth day as PRC.

**Figure 2:**
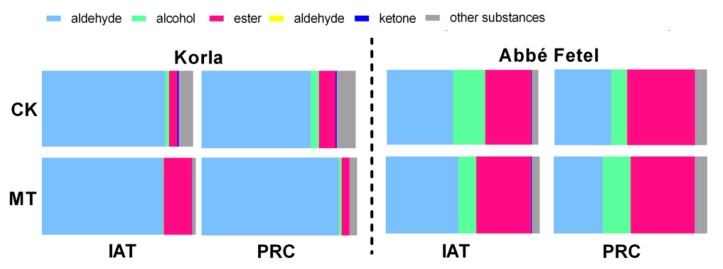
Effect of melatonin on the relative content of volatile compounds classes in postharvest fruit.

Aldehydes were the principal aroma components in ‘Korla’ pear (Figure 2), representing 81.24% and 78.26% in the CK and MT treatment, respectively, following treatment; after storage at 25 °C for three days, the proportion of aldehydes in the MT treatment remained similar (80.11%), but had decreased to 70.70% in the CK. Proportion of esters was higher in the MT treatment than in the CK IAT, and the proportion of alcohol esters in ‘Abbé Fetel’ was greater than that in ‘Korla’. In ‘Abbé Fetel’, the proportion of aldehydes decreased at PRC, while that of esters increased. The proportion of esters was higher IAT in the MT treatment, while the proportion of alcohols at PRC was greater than in the CK.

Hexanal was the dominant volatile recorded in both pear varieties (Table 1). Following treatment, hexanal content of ‘Korla’ IAT was lower in the MT treatment than in the CK; there was no treatment difference in ‘Abbé Fetel’. At PRC, content of hexanal in MT treated ‘Korla’ pear was greater than in the CK, while in ‘Abbé Fetel’ it remained lower than in the CK. The trend in content of (*E*)-hex-2-enal was similar to that of hexanal. MT increased ethyl ester content in ‘Korla’. IAT, MT had inhibited almost all volatile substances, but by the respiratory jump phase at PRC, content of propyl acetate, and hexyl ester had increased (Table 1).

### 2.2. Effects of MT on Linoleic Acid and Linolenic Acid Content

**Figure 3:**
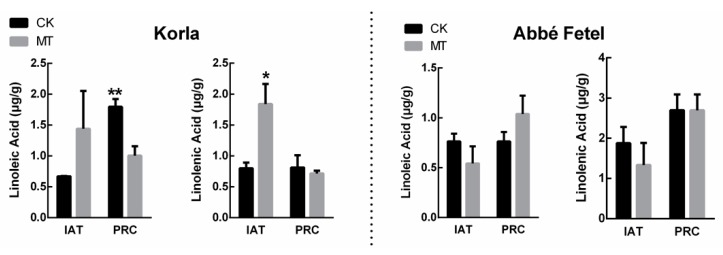
Effect of melatonin on linoleic acid and linolenic acid content. Data are means ±SE of three replicates. * *p* < 0.05, ** *p* < 0.01.

Linolenic acid and linoleic acid are the precursors of the main aromatic substances in the fruit. Content of linolenic acid was higher in MT-treated ‘Korla’ than that in the CK IAT, while at PRC, linoleic acid content was lower than that in the CK. Content of linoleic and linolenic acid in ‘Abbé Fetel’ increased with time, but there was no effect of treatment (Figure 3). 

### 2.3. Effects of MT on Ester-related Enzyme Activity 

**Figure 4:**
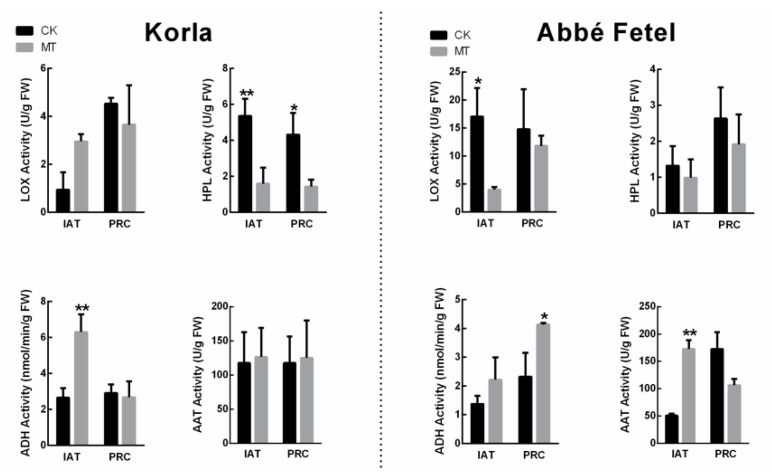
Effect of melatonin on LOX, HPL, ADH, and AAT activity. Data are means ± SE of three replicates. * *p* < 0.05, ** *p* < 0.01.

LOX, HPL, ADH and AAT are key enzymes in the synthesis of aromatic substances. In ‘Korla’, the greatest change in enzyme activity was that of the HPL, which decreased following treatment with MT, while ADH enzyme activity increased following MT treatment IAT. In ‘Korla’, there was no significant difference in LOX and AAT. However, in ‘Abbé Fetel’, LOX enzyme activity was lower in the MT treatment than in the CK IAT, while activity of AAT increased (Figure 4). In ‘Abbé Fetel’, there was a significant increase in ADH activity at PRC in MT group, while there was no significant difference in HPL activity.

### 2.4. Effects of Melatonin on Expression of Ester-related Genes

**Figure 5:**
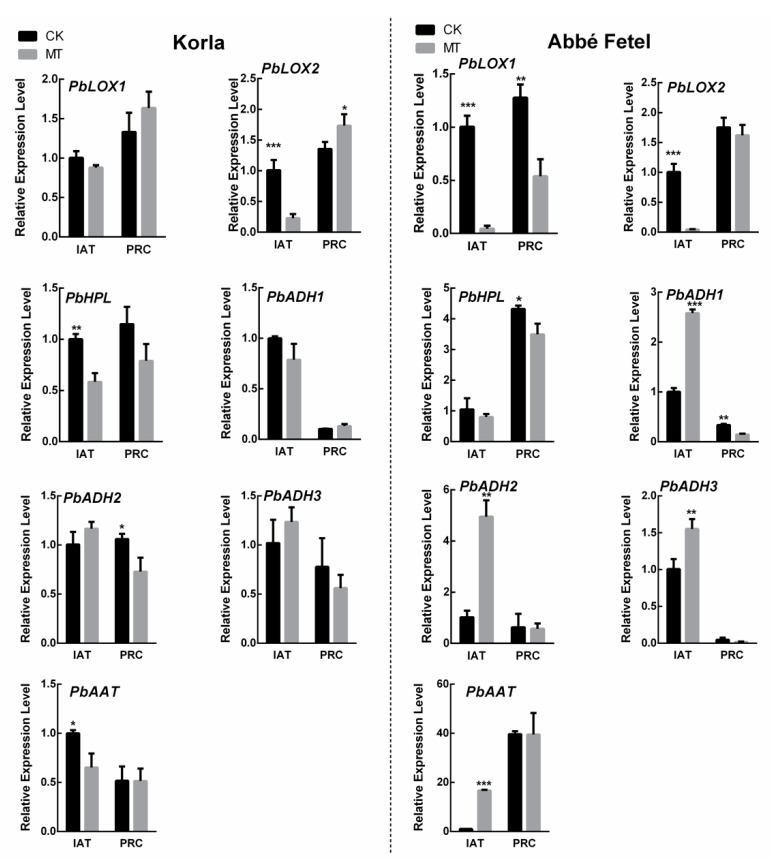
Effect of melatonin on relative expression of ester-related gene. Levels of gene expression at the first sampling point under control conditions was normalized as 1.0. Data are means ± SD of three replicates. * *p* < 0.05, ** *p* < 0.01, *** *p* < 0.001.

q-PCR was used to analyze the expression of the main enzyme encoding genes for aroma synthesis. We found that MT downregulated *PbLOX2* IAT in the two varieties, and downregulated *PbLOX1* in ‘Abbé Fetel’ IAT and at PRC. In ‘Korla’, *PbHPL* was upregulated by MT IAT, while in ‘Abbé Fetel’, MT upregulated *PbHPL* at PRC. In ‘Abbé Fetel’, MT upregulated *PbADH1*, *PbADH2, PbADH3*, and *PbAAT* IAT. However, this difference was not significant in ‘Korla’, which also proved that the increased activity of AAT and ADH was regulated by the encoding enzyme genes (Figure 5).

## 3. Discussion

Numerous studies of effects of MT in a range of postharvest fruit during storage, including peach [18], strawberry [19], litchi [20], and pear [7,21], have shown it inhibits ethylene synthesis, scavenges excessive ROS, retards fruit senescence, and maintains fruit quality. Although it is known that aroma compounds are closely related to ethylene production in postharvest fruit, their response to MT was unclear.

In this study and regardless of treatment, we found 10 volatile compounds dominated in ‘Korla’ pear. Among them, esters are ethyl acetate; ethyl butyrate; butyl acetate; hexyl acetate; alcohols are hex-2-yn-1-ol; hexan-1-ol; aldehydes are hexanal; (*E*)-hex-2-enal; ketones are 6-methylhept-5-en-2-one. Fifteen dominated in ‘Abbé Fetel’ pear IAT; among them, esters are ethyl acetate; propyl acetate; butyl acetate; 2-methylbutyl acetate; pentyl acetate; hexyl acetate; heptyl formate; alcohols are hex-2-yn-1-ol; 2-methylbutan-1-ol; hexan-1-ol; aldehydes are acetaldehyde; hexanal; 2-methylPent-4-enal; (*E*)-hex-2-enal; ketones is 6-methylhept-5-en-2-one. At PRC, content of four volatiles (2-methylpent-4-enal; 6-methylhept-5-en-2-one) were not detected (Table 1). Content of esters were greater, and aldehydes were lower, respectively, in ‘Abbé Fetel’ than in ‘Korla’ IAT. At PRC, content of esters generally increased in ‘Abbé Fetel’, but there was no change in volatile content of ‘Korla’ IAT or at PRC. These results indicate that aroma contents of ‘Abbé Fetel’ pear increase during ripening, whereas those of ‘Korla’ pear remain stable during storage. 

When we analyzed the effect of MT on the aroma components in the two varieties of pear, we found they were dominated by hexanal and (*E*)-hex-2-enal, supporting previous research that similarly showed hexanal is the principal volatile in pear fruit [22]. Among the volatile compounds in pear fruit, C_6_ aldehydes and alcohols are characterized as green-note volatiles, and following treatment with MT, content of hexanal and (*E*)-hex-2-enal decreased in the two varieties. We showed that the fatty acid pathway produced C_6_ aldehydes and alcohols, indicated by linoleic and linolenic acids as precursors catalyzed by LOX. In ‘Korla’, MT increased content of linolenic and linoleic acids, but decreased content of hexanal. Although there was no difference shown in LOX activity, melatonin significantly downregulated HPL activity, indicating MT had reduced oxidation of the fatty acids that led to the lowered hexanal content. Although MT downregulated the *PbLOX2* gene, there was no change in LOX activity in ‘Korla’ pear. HPL is known to catalyze the fatty acid hydroperoxide to generate hexanal and hexenal [23], and we found that HPL activity was inhibited by MT, while expression of *PbHPL* was downregulated IAT. These results indicate that MT regulates biosynthesis of aldehyde in ‘Korla’ pear by downregulating expression of HPL.

In contrast, MT reduced content of (*E*)-hex-2-enal, reduced LOX activity, and downregulated *PbLOX1* and *PbLOX2* in ‘Abbé Fetel’ pear IAT, but there was no effect on content of linoleic and linolenic acids, HPL enzyme activity, or *PbHPL* gene expression. These results indicate that MT regulates content of (*E*)-hex-2-enal and hexanal by inhibiting LOX activity in ‘Abbé Fetel’ pear. Although MT effects on (*E*)-hex-2-enal and hexanal were consistent between ‘Korla’ and ‘Abbé Fetel’, the mechanisms differed, possibly due to variation in ethylene release driven by LOX activity that increases with ripening in ethylene-induced fruits; an increase in enzyme activity is a precursor to an increase in endogenous ethylene in climacteric fruits [24].

We found that MT increased hexyl acetate content in ‘Abbé Fetel’ at PRC. ADH is a different class of green-note volatiles to C_6_ aldehydes and alcohols and is considered a pivotal enzyme in the reduction reaction that transforms C_6_ aldehydes into C_6_ alcohols [25]. With the exception of C_6_ aldehydes and alcohols, esters and lactones are both recognized as fruit-note volatiles that impact on pear fruit aroma. Synthesis of esters derives from the esterification reaction between alcohol and acyl-CoA catalyzed by AAT [26,27], and genes encoding LOX and AAT are dependent on, and regulated by ethylene in apple fruit [28]. Zhou et al. [29] found that volatile esters dominate in ‘Nanguoli’ pear (*Pyrus ussuriensis* Maxim.), while higher ester content has been found in fruit treated with intermittent warming compared with 1-MCP treated fruit. Ethylene is positively related to ester content; however, in our study, while MT inhibited ethylene production, there was no effect on esters. The reason was that although melatonin inhibited ethylene production, it regulated the enzyme activity associated with aroma synthesis, activated activity of ADH and AAT, and promoted the conversion of alcohols to esters which ultimately increases content of esters in fruit. This is the main reason why the amount of aldehydes decreased after melatonin treatment, but the content of esters increased.

**Figure 6:**
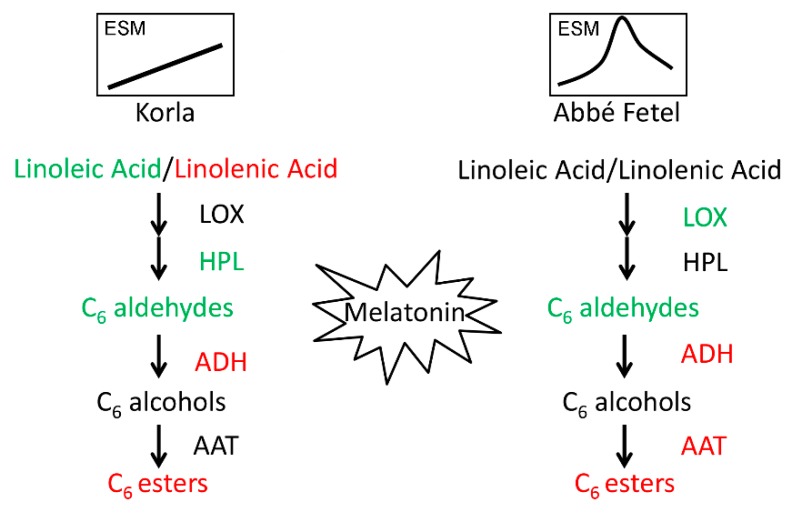
Model depicting the mechanism of the effect of melatonin on aroma in ‘Korla’ and ‘Abbé Fetel’. Red: upregulation; Green: downregulation. ESM: Synthesis model of ethylene in postharvest fruits.

In summary, we found the principal aromatic components in ‘Korla’ and ‘Abbé Fetel’ pear varieties were the C_6_ aldehydes, alcohols, and esters. In ‘Korla’, MT inhibited HPL activity and downregulated *PbHPL* expression to reduce content of hexanal and (*E*)-hex-2-enal, whereas in ‘Abbé Fetel’, MT inhibited LOX enzyme activity, downregulated expression of *PbLOX1* and *PbLOX2*, and promoted activity of ADH and AAT that similarly reduced content of hexanal and (*E*)-hex-2-enal, and increased the content of propyl acetate, and hexyl acetate (Figure 6). This study lays a foundation for the application of MT in the effective storage and preservation of postharvest fruit.

## 4. Materials and Methods

### 4.1. Plant Material and Treatments

‘Korla’ (*Pyrus brestschneideri* Rehd) and ‘Abbé Fetel’ (*Pyrus communis* L.) pear fruit were harvested from commercial orchards in Dali County, Shaanxi Province and Wugong County, Shaanxi Province, China, respectively, at commercial maturity on 3 September 2018. Treatments comprised immersion of 90 fruit of each variety in distilled water as the negative control (CK) or 100 μmol L^−1^ MT distilled water solution (Wako, Wako Pure Chemical Industries, Ltd., Chuo-ku, Japan) at 25 °C for 12 h. ‘Korla’ fruit were treated immediately after harvest, whereas ‘Abbé Fetel’ fruit were initially stored at 4 °C and >95% relative humidity for 1 month, and treated subsequently. Following treatment, five replicates of 18 fruit per treatment were placed in sealed plastic bags, and stored at 25 °C. Treatment effects on all variables were measured immediately after treatment (IAT) and at the climacteric respiration peak of the CK fruit (PRC), unless stated otherwise. 

### 4.2. Fruit Volatiles

Volatile compounds were determined using GC-MS based on solid-phase micro-extraction (SPME), according to a modified method reported by Zhang et al [30]. We used a four point method to extract juice from the pear fruit, where 10 mL of pear juice and 3.6 g of NaCl were equilibrated at 40 °C for 10 min in a sample bottle, prior to extraction of volatile compounds using a fiber coated with 65 μm of polydimethylsiloxane and divinylbenzene (PDMS/DVB) (Supelco, Bellefonte, PA, USA) at 40 °C for 40 min. The adsorbed volatiles on the extraction head were then desorbed for 2 min at 250 °C into the splitless injection port of the GC, and volatile compounds were analyzed using a GC-MS (ISQ & TRACE ISQ, Thermo Fisher Scientific, Waltham, MA., USA) fitted with a 60 m × 0.32 mm × 1 μm elastic quartz capillary column (Thermo Fisher Scientific, Waltham, MA, USA), with He carrier gas (99.99%), no diffluence, and a programmed temperature rise, with an inlet temperature of 250 °C. The oven temperature was held at 50 °C for 1 min, then it increased by 5 °C min^−1^ to 120 °C, 8 °C min^−1^ to 200 °C, and finally by 12 °C min^−1^ to 250 °C. Electronic ionization was used at 70 eV, and detection was performed in full scan mode, from 29 to 600 amu. Percentage peak area was used to describe relative content. The internal standard was 2-nonanone. The linear regression range, equations of the standard curves, regression coefficients (R^2^), and correction factor (CF) for each volatile standard are listed in Table S1.

### 4.3. Ethylene Production 

Ethylene production from three replicates of 10 fruit per treatment was measured daily until the CK fruit had decayed, following the method described by Xie et al [31].

### 4.4. Linoleic and Linolenic Acid Content

A sample of 0.5 g of fruit from each treatment was ground in a pre-cooled mortar with 1 mL of precooling reagent that comprised petroleum ether; the sample was then transferred to a new tube for ultrasound extraction for 40 min and centrifugation at 10,000× *g* for 10 min. Supernatant was extracted, and residues were treated with 0.5 mL petroleum ether, before ultrasound extraction for 20 min and centrifugation at 10,000× *g* for 10 min. Two supernatants were combined and dried using N_2_, diluted to fixed volume (0.5 mL) with methanol, and then the solution was filtered into a sample bottle. The HPLC (L-3000, Rigol, Beijing, China) liquid phase used a chromatographic column C18 (4.6 nm × 250 mm × 5 μm, Kromasil, AKZO NOBEL, Västra Götaland, Sweden), and the mobile phase conditions comprised 80:20 ratio of acetonitrile to 0.1% phosphoric acid, 204 nm wave length, and a column temperature of 30 °C.

### 4.5. Enzyme Activity 

LOX enzymes were extracted from 0.1 g of fruit tissue ground in liquid nitrogen using polyvinylpolypyrrolidone (PVPP), to which 1 mL of enzyme extract (0.1 mM phosphate buffer, pH = 7.5, 2 mM DTT, 1 mM disodium EDTA, and 0.1% (*v*/*v*) Triton X-100) was added, and then the mixture was centrifuged for 15 min at 4 °C and 15,000× *g*. The reaction system comprised 0.1 mM phosphate buffer (pH = 7.5), reaction substrate solution (8.6 mM linoleic acid and 0.25% (*v*/*v*) Tween-20 soluble in 0.1 mM phosphate buffer), and crude enzyme solution. Change in absorbance per min at OD_280_ was recorded 15 s after the addition of the enzyme solution, where each change in OD value was denoted as 1 U [32].

HPL activity was determined following a modified version of the approach described by Vick [33], where 3 g of fruit tissue was treated with 6 mL of pre-cooled (4 °C) extracting solution that contained 150 mmol/L HEPES-KOH buffer (pH = 8.0), 250 mmol L^-1^ sorbitol, 10 mmol L^−1^ EDTA, 10 mmol L^−1^ MgCl_2_, 1% (*v*/*v*) glycerol, 4% PVPP, and 0.1mmol L^−1^ MPMSF (protease inhibitor). Then, samples were ground using a pre-chilled mortar and pestle, fully blended, and centrifuged at 15,000× *g* (4 °C) for 30 min. HPL activity was determined using a 3.5 mL reaction system, comprising 2 mL of analytical buffer (150 mmol L^−1^ HEPES-KOH, pH = 8.0; 250 mmol L^−1^ sorbitol; 10 mmol L^−1^ EDTA; and, 10 mmol L^-1^ MgCl_2_), 0.75 mL of reaction substrate, 0.15 mL of 1.6 mmol L^−1^ NADH, 0.1 mL of ADH enzyme extract (1.5 mg/3 mL phosphate buffer, pH = 8.6), and 0.5 mL of crude enzyme supernatant, and determined at a reaction temperature of 30 °C and wavelength of 340 nm. Sodium hydroperoxide linoleate was used as the substrate for determination of enzyme activity, and the reaction substrate comprised 10 mL of distilled water, 200 μL of 10 mmol L^−1^ sodium linoleate, 400 μL of LOX enzyme extract (1 mg/10 mL phosphate buffer, pH = 9.0) that was heated in a water bath for 2 h at 30 °C. Change in absorbance per min at OD_340_ was recorded 15 s after addition of the enzyme solution, where each change in OD value was denoted as 1 U [32]. Enzyme activity was measured three times per sample.

The method of extraction of ADH enzymes was similar to that of LOX enzymes, where the reaction system comprised 0.15 mM NADH, 80 mM acetaldehyde, and enzyme extract. Changes in absorbance per min at OD_340_ were recorded.

AAT acyltransferase activity was determined using a slightly modified version of the method described by EcheverrıÁ et al. [25], where 3.0 g samples of fruit were ground in 0.1 g PVPP using a pre-chilled mortar and pestle, homogenized in 6.0 mL of 0.1 mol/L potassium phosphate buffer (pH = 7.0), and centrifuged at 12,000× *g* (4 °C) for 30 min. The reaction system, which comprised 0.5 mol·L^−1^ Tris-HCl buffer (pH = 7.0), 11.6 mmol·L^−1^ MgCl_2_, 0.3 mmol·L^−1^ acetyl-CoA, 10 mmol·L^−1^ butyl alcohol, and 0.6 mL of supernatant, was maintained at 35 °C for 15 min, before 150 μL of 20 mmol·L^−1^ dithio-bis-nitrobenzoic acid was added and kept at room temperature for 10 min. Then, AAT activity was measured at 412 nm 15 s after the enzyme solution had been added; changes in absorbance per 1 min at OD_412_ were recorded, where each change in OD value was denoted as 1 U (Axelrod et al., 1981).

### 4.6. Gene Expression

Levels of expression of related genes were assessed as three biological replicates of 5 g composite samples of flesh taken from three fruit in each of the five replicates at the two time points (IAT and PRC); samples were frozen in liquid nitrogen and stored at −80 °C prior to analysis.

Total RNA was purified using an RNAprep Pure Plant Kit (Tiangen, Beijing, China), and RNA concentration was detected using UV spectrophotometry. Then, 1 mg of total RNA was reverse-transcribed to cDNA using a PrimeScript RTreagent kit with gDNA Eraser (TaKaRa, Dalian, China). Three replicates of qRT-PCR reactions were performed on an Icycler iQ5 (Bio-Rad, Berkeley, CA, USA) with SYBR Premix Ex Taq II (TaKaRa, Dalian, China), according to the manufacturer’s instructions. Data were analyzed using iQ5 2.0 software (Bio-Rad, Berkeley, CA, USA) with the ddCT algorithm.

DNA coding sequences of candidate genes for RT-PCR were isolated from the pear genome (https://www.ncbi.nlm.nih.gov/genome/12793) using BLAST searches of published sequences. For lipoxygenase genes, linoleate 13S-lipoxygenase 2-1 (XM_009356413.2) and linoleate 13S-lipoxygenase 2-1 (XM_009366759.2) were amplified and designated as *PbLOX1* and *PbLOX2*, respectively; for fatty acid hydroperoxide lyase genes, fatty acid hydroperoxide lyase (XM_009348862.2) was amplified and designated as *PbHPL*; for alcohol dehydrogenase genes, alcohol dehydrogenase (XM_009362449.1), alcohol dehydrogenase-like 1 (XM_009350668.2), and alcohol dehydrogenase-like (XM_009374145.2) were amplified and designated as *PbADH1*, *PbADH2*, and *PbADH3*, respectively; and, for alcohol acyltransferase, methanol O-anthraniloyltransferase-like 1 (XM_018649523.1) was amplified and designated as *PbAAT*. Primers and references for *actin*, lipoxygenase genes, fatty acid hydroperoxide lyase genes, alcohol dehydrogenase genes, and alcohol acyltransferase related genes are listed in Table S2.

### 4.7. Statistical Analysis

Treatment differences were tested using the Student’s t test, at *p* < 0.05 in SPSS 23.0. Results are presented as means ± SDs of at least three replicate samples.

## Figures and Tables

**Table 1:** Effects of melatonin on content of volatile compounds (μg/kg) in postharvest ‘Korla’ and ‘Abbé Fetel’ pears.

RT	Compound Name	Cas #	Molecular Formula	Korla Volatile Contents (μg/kg)				Abbé Fetel Volatile Contents (μg/kg)			
				IAT		PRC		IAT		PRC	
				CK	MT	CK	MT	CK	MT	CK	MT
4.18	Acetaldehyde ^b^	75-07-0	C_2_H_4_O	-	-	-	-	0.558 ± 0.229	0.287 ± 0.109	1.045 ± 0.017	1.095 ± 0.273
5.51	Ethyl Acetate ^a^	141-78-6	C_4_H_8_O_2_	1.44 ± 0.406	5.87 ± 1.895	1.45 ± 0.544	8.74 * ± 2.756	2.37 ± 0.500	2.04 ± 1.12	6.54 ± 0.370	4.955 ± 1.06
6.69	n-Propyl acetate ^a^	109-60-4	C_5_H_10_O_2_	-	-	-	-	0.670 * ± 0.188	0.207 ± 0.022	1.20 ± 0.281	3.81 * ± 1.11
7.82	Butanoic acid, ethyl ester ^a^	105-54-4	C_6_H_12_O_2_	1.51 ± 0.263	8.02 * ± 1.320	1.52 ± 0.060	4.68 ± 2.766	-	-	-	-
8.50	Acetic acid, butyl ester ^a^	123-86-4	C_6_H_12_O_2_	0.417 ± 0.141	0.373 ± 0.150	0.461 ± 0.074	0.340 ± 0.179	3.56 ± 0.839	2.37 ± 0.338	16.58 ± 1.94	10.12 ± 3.87
8.78	Hexanal ^a^	66-25-1	C_6_H_12_O	225.30 * ± 33.18	150.84 ± 29.58	201.03 ± 75.47	230.80 ± 34.22	45.57 ± 8.36	36.70 ± 8.83	102.40 ± 27.58	62.91 ± 18.64
9.63	1-Butanol, 2-methyl-, acetate ^a^	624-41-9	C_7_H_14_O_2_	-	-	-	-	2.24 ± 0.665	0.877 ± 0.330	10.57 ± 1.31	14.61 ± 6.64
10.11	4-Pentenal, 2-methyl- ^b^	5187-71-3	C_6_H_10_O	0.151 ± 0.0284	0.262 ± 0.197	0.161 ± 0.062	0.564 ± 0.302	0.197 ± 0.0499	0.133 ± 0.0158	-	-
10.90	Acetic acid, pentyl ester ^a^	628-63-7	C_7_H_14_O_2_	-	-	-	-	2.26 ± 1.03	2.41 ± 0.355	17.00 ± 5.06	18.499 ± 3.26
11.80	2-Hexyn-1-ol ^b^	764-60-3	C_6_H_10_O	0.414 ± 0.030	0.773 ± 0.439	0.474 ± 0.196	1.352 ± 0.459	0.251 ± 0.0792	0.253 ± 0.0379	0.311 ± 0.109	0.116 ± 0.0803
12.39	2-Hexenal ^a^	505-57-7	C_6_H_10_O	19.51 ± 6.29	16.25 ± 1.33	12.61 ± 2.37	17.60 ± 0.469	13.25 ** ± 0.696	9.168 ± 0.249	23.67 ** ± 8.87	7.25 ± 1.10
12.64	1-Butanol, 2-methyl- ^a^	137-32-6	C_5_H_12_O	-	-	-	-	0.577 ± 0.0971	0.108 ± 0.0380	1.49 *** ± 0.0495	0.167 ± 0.0170
13.52	Acetic acid, hexyl ester ^a^	142-92-7	C_8_H_16_O_2_	1.87 ± 0.796	4.58 ± 1.619	6.72 ± 1.017	3.10 ± 0.770	20.14 ± 5.47	14.21 ± 6.58	76.22 ± 0.488	123.91 ** ± 15.24
15.36	5-Hepten-2-one, 6-methyl- ^b^	110-93-0	C_8_H_14_O	0.112 ± 0.005	0.336 ± 0.164	0.207 ± 0.091	0.187 ± 0.024	0.238 ± 0.0344	0.390 ± 0.220	-	-
16.50	1-Hexanol ^a^	111-27-3	C_6_H_14_O	0.043 ± 0.020	0.021 ± 0.013	0.016 ± 0.008	0.025 ± 0.011	0.780 ± 0.00552	0.678 ± 0.346	0.612 ± 0.0795	0.417 ± 0.310
18.93	Formic acid, heptyl ester ^b^	112-23-2	C_8_H_16_O_2_	-	-	-	-	0.0180 ± 0.00572	0.692 ± 0.0382	0.0496 ± 0.0197	0.0584 ± 0.00119

^a^ Substances determined after calibration by external standard and internal standard. ^b^ relative content determined using the internal standard method. * *p* < 0.05, ** *p* < 0.01, *** *p* < 0.001.

## Supplementary Materials

Figure S1: GC-MS chromatograms of volatile production determinations, Table S1: Authentic standard compounds used in the GC-MS analysis and standard curve equations, Table S2: List of qRT-PCR primers.

## References

[1] Arnao M.B., Hernández-Ruiz J. (2014). Melatonin: Plant growth regulator and/or biostimulator during stress?. Trends Plant Sci..

[2] Arnao M.B., Hernández-Ruiz J. (2015). Functions of melatonin in plants: A review. J. Pineal Res..

[3] Arnao M.B., Hernández-Ruiz J. (2019). Melatonin: A new plant hormone and/or a plant master regulator?. Trends Plant Sci..

[4] Kanwar M.K., Yu J., Zhou J. (2018). Phytomelatonin: Recent advances and future prospects. J. Pineal Res..

[5] Sharif R., Xie C., Zhang H., Arnao M.B., Ali M., Ali Q., Muhammad I., Shalmani A., Azher N.M., Chen P. (2018). Melatonin and its effects on plant systems. Molecules.

[6] Wang Y., Reiter R.J., Chan Z. (2018). Phytomelatonin: A universal abiotic stress regulator. J. Exp. Bot..

[7] Zhai R., Liu J., Liu F., Zhao Y., Liu L., Fang C., Wang H., Li X., Wang Z., Ma F. (2018). Melatonin limited ethylene production, softening and reduced physiology disorder in pear (*Pyrus communis* L.) fruit during senescence. Postharvest Biol. Technol..

[8] Meng J., Yu Y., Shi T., Fu Y., Zhao T., Zhang Z. (2018). Melatonin treatment of pre-veraison grape berries modifies phenolic components and antioxidant activity of grapes and wine. Food Sci. Tech-Braz..

[9] Hu W., Tie W., Ou W., Yan Y., Kong H., Zuo J., Ding X., Ding Z., Liu Y., Wu C. (2018). Crosstalk between calcium and melatonin affects postharvest physiological deterioration and quality loss in cassava. Postharvest Biol. Technol..

[10] Sun Q., Zhang N., Wang J., Zhang H., Li D., Shi J., Li R., Weeda S., Zhao B., Ren S. (2014). Melatonin promotes ripening and improves quality of tomato fruit during postharvest life. J. Exp. Bot..

[11] Zhang B., Yin X., Li X., Yang S., Ferguson I.B., Chen K. (2009). Lipoxygenase gene expression in ripening kiwifruit in relation to ethylene and aroma production. J. Agric. Food Chem..

[12] Schaffer R.J., Friel E.N., Souleyre E.J.F., Bolitho K., Thodey K., Ledger S., Bowen J.H., Ma J.H., Nain B., Cohen D. (2007). A genomics approach reveals that aroma production in apple is controlled by ethylene predominantly at the final step in each biosynthetic pathway. Plant Physiol..

[13] Yang X.T., Song J., Du L.N., Forney C., Leslie C.P., Sherry F., Wismer P., Zhang Z.Q. (2016). Ethylene and 1-MCP regulate major volatile biosynthetic pathways in apple fruit. Food Chem..

[14] Lia G.P., Jia H.J., Li J.H., Li H.X., Teng Y.W. (2016). Effects of 1-MCP on volatile production and transcription of ester biosynthesis related genes under cold storage in ‘Ruanerli’ pear fruit (*Pyrus ussuriensis* Maxim.). Postharvest Biol. Technol..

[15] Wang B.H., Sun X.X., Dong F.Y., Zhang F., Niu J.X. (2014). Cloning and expression analysis of an MYB gene associated with calyx persistence in Korla fragrant pear. Plant Cell Rep..

[16] Schwab W., Davidovich-Rikanati R., Lewinsohn E. (2008). Biosynthesis of plant-derived flavor compounds. Plant J. Cell Mol. Biol..

[17] Manríquez D., El-Sharkawy I., Flores F.B., El-Yahyaoui F., Regad F., Bouzayen M., Latché A., Pech J.C. (2006). Two highly divergent alcohol dehydrogenases of melon exhibit fruit ripening-specific expression and distinct biochemical characteristics. Plant Mol. Biol..

[18] Gao H., Zhang Z., Chai H., Cheng N., Yang Y., Wang D., Yang T., Cao W. (2016). Melatonin treatment delays postharvest senescence and regulates reactive oxygen species metabolism in peach fruit. Postharvest Biol. Technol..

[19] Liu C., Zheng H., Sheng K., Liu W., Zheng L. (2018). Effects of melatonin treatment on the postharvest quality of strawberry fruit. Postharvest Biol. Technol..

[20] Zhang Y., Huber D.J., Hu M., Jiang G., Gao Z., Xu X., Jiang Y., Zhang Z. (2018). Delay of postharvest browning in litchi fruit by melatonin via the enhancing of antioxidative processes and oxidation repair. J. Agric. Food Chem..

[21] Liu J., Jie Y., Zhang H., Cong L., Zhai R., Yang C., Wang Z., Ma F., Xu L. (2019). Melatonin inhibits ethylene synthesis via nitric oxide regulation to delay postharvest senescence in pears. J. Agric. Food Chem..

[22] Tian H.L., Zhan P., Deng Z.Y., Yan H.Y., Zhu X.R. (2014). Development of a flavour fingerprint by GC-MS and GC-O combined with chemometric methods for the quality control of Korla pear (*Pyrus serotina* Reld). Int. J. Food Sci. Tech..

[23] Rapparini F., Predieris S. (2003). Pear fruit volatiles. Hortic. Rev..

[24] Gomez-Lobato M.E., Civello P.M., Martínez G.A. (2012). Expression of a lipoxygenase encoding gene (BoLOX1) during postharvest senescence of broccoli. Postharvest Biol. Technol..

[25] EcheverrıÁ G., Graell J., López M.L., Lara I. (2004). Volatile production, quality andaroma-related enzyme activities during maturation of ‘Fuji’ apples. Postharvest Biol. Technol..

[26] Becerra Guerrero J., Rodríguez-Palacios A. (2004). Functional characterization of enzymes forming volatile esters from strawberry and banana. Plant Physiol..

[27] Souleyre E.J., Greenwood D.R., Friel E.N., Karunairetnam S., Newcomb R.D. (2005). An alcohol acyl transferase from apple (cv. Royal Gala), MpAAT1, produces esters involved in apple fruit flavor. FEBS J..

[28] Defilippi B.G., Kader A.A., Dandekar A.M. (2005). Apple aroma: Alcohol acyltransferase, a rate limiting step for ester biosynthesis, is regulated by ethylene. Plant Sci..

[29] Zhou X., Dong L., Zhou Q., Wang J., Chang N., Liu Z., Ji S. (2015). Effects of intermittent warming on aroma-related esters of 1-methylcyclopropene-treated ‘Nanguo’ pears during ripening at room temperature. Sci. Hortic..

[30] Zhang B., Shen J.Y., Wei W.W., Xi W.P., Xu C.J., Ferguson I., Chen K. (2010). Expression of genes associated with aroma formation derived from the fatty acid pathway during peach fruit ripening. J. Agric. Food Chem..

[31] Xie X., Einhorn T., Wang Y. (2015). Inhibition of ethylene biosynthesis and associated gene expression by aminoethoxyvinylglycine and 1-methylcyclopropene and their consequences on eating quality and internal browning of ‘Starkrimson’ pears. J. Am. Soc. Hortic. Sci..

[32] Axelrod B., Cheesbrough T.M., Laakso S., John M.L. (1981). Lipoxygenase from soybeans: Ec 1.13.11.12 linoleate:oxygen oxidoreductase. Method. Enzym..

[33] Vick B.A. (1991). A spectrophotometric assay for hyderperoxidelyase. Lipids.

