# Supplementary material for: Effects of Melatonin Treatment of Postharvest Pear Fruit on Aromatic Volatile Biosynthesis
Source: Molecules. 2019 Nov 21;24(23):4233. doi: 10.3390/molecules24234233 (PMC6930639; doi:10.3390/molecules24234233)
Supplement: Supplementary file 1 [file molecules-24-04233-s001.pdf]

Table S1. Authentic standard compounds used in the GC-MS analysis and standard curve equations.

Chemical	Source[a]	Range (µg/mL)	Curves[b]	CF[c]	R^2
Ethyl Acetate	Sigma	0.009-0.036	y = 6,074,673,634.05 x + 64,447,384.87	2.29	0.974
Propyl acetate	Sigma	0.00875-0.035	y = 9,083,131,385.96 x + 217,940,374.05	2.15	0.989
Ethyl butyrate	Sigma	0.00875-0.035	y = 22,875,292,640.97 x + 57,135,891.73	3.43	0.99
Butyl acetate	Sigma	0.00875-0.035	y = 7,382,735,532.07 x + 130,891,812.94	0.76	0.991
Hexanal[a]	Sigma	0.00825-0.035	y = 8,450,347,240.69 x + 114,964,167.33	5.2	0.991
2-Methylbutyl acetate	Sigma	0.0035-0.00875	y = 17,096,133,241.01 x + 59,925,828.39	2.34	0.996
Amyl acetate	Sigma	0.00875-0.035	y = 23,152,246,097.23 x - 39,201,024.73	2.66	0.996
Hex-2-Enal	Sigma	0.00825-0.035	y = 16,276,075,224.10 x - 132,779,325.15	1	0.990
2-Methyl-1-butanol	Sigma	0.016-0.048	y = 3,905,760,970.87 x - 27,960,897.71	0.176	0.986
Hexyl acetate	Sigma	0.0085-0.034	y = 23,468,843,104.46 x + 114,015,306.80	3	0.995
Hexyl alcohol	Sigma	0.016-0.048	y = 2,657,296,400.25 x - 4,202,342.98	0.11	0.997

[a]Sigma, Sigma-Aldrich Co. Ltd. (St. Louis, MO, USA);
[b]y is the ratio of the area of the peak of an authentic standard; x is the weight of an authentic standard in µg.
[c]CF: correction factor. CF was calculated as a mean using [(C1/A1)/(C2/A2), where C1 is the concentration of the authentic standard, A1 is the peak area of the authentic standard (TIC) or the peak area of the corresponding quantifying ion (SIM mode), C2 is the concentration of the internal standard, and A2 is the peak area of the quantifying ion of the internal standard.

Table S2. List of qRT-PCR primers

Primers	Forward/Reverse	Sequences(5'-3')
PbLOX1	F	CCTCCCTGATCAGCATCGTT
	R	TTTGAGCTCACCAGAAGCGG
PbLOX2	F	GGTGATCCGTGCCACTTCTA
	R	AACCCTCCTGTTACTGTCACC
PbHPL	F	CCGGACACCTTTAAACCGGA
	R	TGGACTCACTTGGTGAACCG
PbADH1	F	GCATTGAATGTACAGGAAGCGTT
	R	GAAGACGGCTTCTTTGTGTGG
PbADH2	F	CGACTGCAACAAGCGTTCAA
	R	GGAAGGTCGGTCTTCGGTTT
PbADH3	F	CACCTCAGGCCATGGAAGTT
	R	TGGCCGAAAATTCGAGGGAA
PbAAT	F	TCCACCCCACCTAATTTCCAC
	R	TGAATGAAATGCGGACATCCTG

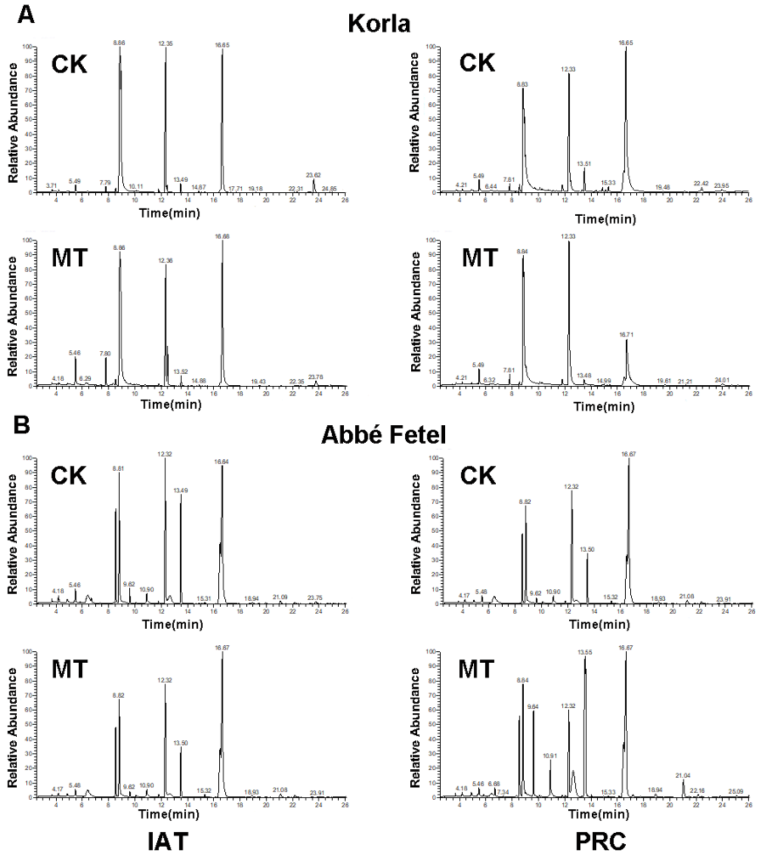

Figure S1. GC-MS chromatograms of volatile production determinations. (a) effect of melatonin on volatile production in 'Korla' pear; (b) effect of melatonin on volatile production in 'Abbé Fetel' pear.
